# Supplementary material for: Identification of photoperiod-induced specific miRNAs in the adrenal glands of Sunite sheep (Ovis aries)
Source: Front Vet Sci. 2022 Jul 22;9:888207. doi: 10.3389/fvets.2022.888207 (PMC9354845; doi:10.3389/fvets.2022.888207)
Supplement: Supplementary file 1 [file Data_Sheet_1.ZIP › Supplementary file/miRDeep2 file.zip!/miRDeep2 file/Know_Structure/SPLP42A1/oar-mir-17.pdf]

miRBase precursor	: oar-mir-17
Total read count	: 3475
oar-miR-17-5p read count	: 3435
remaining reads	: 40

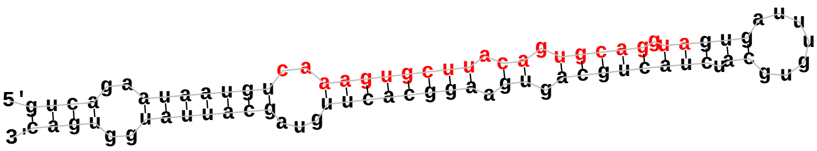

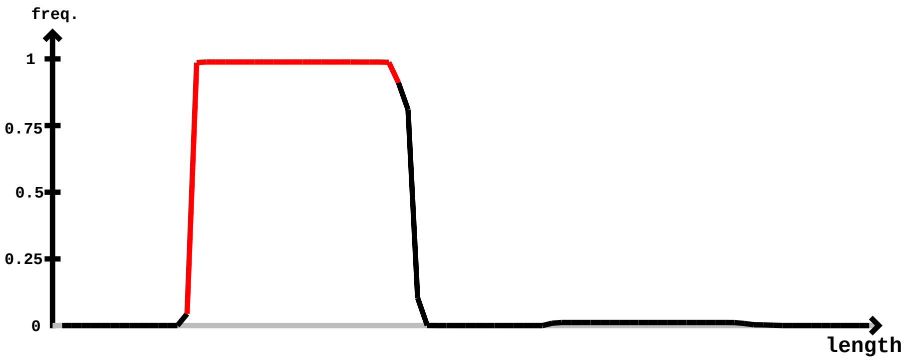


```
                 oar-miR-17-5p
5'- gucagaauaaugucaaagugcuuacagugcagguagugauuugugcaucuacugcagugaaggcacuuguagcauuauggugac  -3'    exp
    ((((..(((((((..((((((((.((.(((((.(((((.......)).)))))))))).)).))))))))...)))))))..))))   reads      mm         sample
    ............ucaaagugcuuacagugcagg...................................................   1          0          seq
    ............ucaaagugcuuacagugcaggu..................................................   32         0          seq
    ............ucaaagugcuuacagugcaggua.................................................   24         0          seq
    ............ucaaagugcuuacagugcagguag................................................   91         0          seq
    ............ucaaagugcuuacagugcagguagu...............................................   5          0          seq
    .............caaagugcuuacagugU......................................................   1          1          seq
    .............caaagugcuuacagugcagg...................................................   3          0          seq
    .............caaagugcuCacagugcaggu..................................................   1          1          seq
    .............caaagugcuuacagugcaggu..................................................   230        0          seq
    .............caaagugcuCacagugcaggua.................................................   3          1          seq
    .............caaagugcuuacagugcaggua.................................................   325        0          seq
    .............caaagugcuuacagugcagguag................................................   2322       0          seq
    .............caaagugcuCacagugcagguag................................................   34         1          seq
    .............caaagugcuuacagugcagguagu...............................................   351        0          seq
    .............caaagugcuCacagugcagguagu...............................................   2          1          seq
    .............caaagugcuuacagugcagguagug..............................................   1          0          seq
    ..............aaagugcuuacagugcaggu..................................................   1          0          seq
    ..............aaagugcuuacagugcaggua.................................................   1          0          seq
    ..............aaagugcuuaUagugcagguag................................................   1          1          seq
    ..............aaagugcuuacagugcagguag................................................   5          0          seq
    ..............aaagugcuuacagugcagguagu...............................................   1          0          seq
    ..................................................acugcagugaaggcacuug...............   1          0          seq
    ..................................................acugcagugaaggcacuugu..............   11         0          seq
    ..................................................acugcagugaaggcacuugua.............   16         0          seq
    ..................................................acugcagugaaggcacuuguag............   2          0          seq
    ..................................................acugcagugaaggcacuuguagc...........   1          0          seq
    ...................................................cugcagugaaggcacuugu..............   1          0          seq
    ...................................................cugcagugaaggcacuuguagc...........   4          0          seq
    ...................................................cugcagugaaggcacuuguagca..........   4          0          seq
```
